# Supplementary material for: Human resistin is critical to activation of the NLRP3 inflammasome in macrophages
Source: PLoS One. 2026 Apr 10;21(4):e0337682. doi: 10.1371/journal.pone.0337682 (PMC13068211; doi:10.1371/journal.pone.0337682)
Supplement: S1 Table — (TIF) [file pone.0337682.s006.docx]

**Supplementary Table 1.** **Primers used for quantitative RT- PCR.**

| **Species** | **Gene query** | **Gene symbol** | **Ref seq number** | **Assay ID** |
| --- | --- | --- | --- | --- |
| Human | BTK | BTK | NM_000061 | Hs.PT.56a.1740934 |
|  | HMGB1 | HMGB1 | NM_002128 | Hs.PT.58.2259017 |
|  | NLRP3 | NLRP3 | NM_001079821 | Hs.PT.58.39303321 |
|  | Caspase-1 | CASP1 | NM_033294 | Hs.PT.56a.22997425.g |
|  | IL1β | IL1B | NM_000576 | Hs.PT.58.1518186 |
|  | IL-18 | IL18 | NM_001243211 | Hs.PT.58.25675872 |
|  | Resistin | RETN | NM_001193374 | Hs.PT.58.20453577.g |
|  | Beta-actin | ACTB | NM_001101 | Hs.PT.39a.22214847 |
| Mouse | BTK | BTK | NM_013482 | Mm.PT.58.7040410 |
|  | HMGB1 | HMGB1 | NM_010439 | Mm.PT.58.32405798.g |
|  | NLRP3 | NLRP3 | NM_145827 | Mm.PT.58.13974318 |
|  | Caspase-1 | CASP1 | NM_009807 | Mm.PT.58.13005595 |
|  | IL1β | IL1B | NM_008361 | Mm.PT.58.41616450 |
|  | IL-18 | IL18 | NM_008360 | Mm.PT.58.42776691 |
|  | RELMα | Retnla | NM_020509 | Mm.PT.58.43062398 |
|  | Beta-actin | Actb | NM_007393 | Mm.PT.39a.22214843.g |
