# Supplementary material for: Human resistin is critical to activation of the NLRP3 inflammasome in macrophages
Source: PLoS One. 2026 Apr 10;21(4):e0337682. doi: 10.1371/journal.pone.0337682 (PMC13068211; doi:10.1371/journal.pone.0337682)
Supplement: S2 Table — (DOCX) [file pone.0337682.s007.docx]

**Supplementary Table 2. Reagents and materials used for this study.**

| **Reagent or resource** | **Resource** | **Catalog #/Identifier** |
| --- | --- | --- |
| **Antibody** |  |  |
| BTK | Sigma-Aldrich | SAB4502936, RRID:AB_10746857 |
| HMGB1 | Abcam | ab79823, RRID:AB_1603373 |
| NLRP3 | Abcam | ab214185, RRID:AB_2819003 |
| NLRP3 | AdipoGen | AG-20B-0014, RRID:AB_2490202 |
| Caspase-1 | Abcam | ab207802, RRID:AB_2889889 |
| IL-1β | Abcam | ab254360, RRID:AB_2936299 |
| IL-18 | Invitrogen | PA5-76082, RRID:AB_2719809 |
| Phospho-BTK | Sigma-Aldrich | SAB4503802 |
| Phospho-tyrosine | Sigma-Aldrich | SAB5600274 |
| Beta-actin | Cell Signaling Technology | 3700 (also 3700P, 3700S), RRID:AB_2242334 |
| p-AKT | Cell Signaling Technology | 4060 (also 4060L, 4060S, 4060T), RRID:AB_2315049 |
| AKT | Cell Signaling Technology | 9272 (also 9272S), RRID:AB_329827 |
| p-ERK1/2 | Cell Signaling Technology | 4370 (also 4370L, 4370S, 4370P, 4370T), RRID:AB_2315112 |
| ERK | Cell Signaling Technology | 4695 (also 4695P, 4695S), RRID:AB_390779 |
| Mouse RELMα | R and D Systems | MAB1523, RRID:AB_2253609 |
| CD79 | Abcam | ab134147 |
| MAC2 | Cedarlane | CL8942AP, RRID:AB_10060357 |
| MPO | R&D Systems | AF3667, RRID:AB_2250866 |
| Human resistin | R&D Systems | AF1359, RRID:AB_416529 |
| Recombinant human IL-1β protein (active) | Abcam | ab259387 |
| Recombinant human IL-18 | R&D Systems | 9124-IL-010 |
| Human IL-1β neutralizing ab | Invivogen | mabg-hil1b-3 |
| Human IL-18 neutralizing ab | R&D Systems | D044-3 |
|  |  |  |
| **Reagents** |  |  |
| Ibrutinib | Selleckchem |  |
| MCC950 | Invivogen | Inh-mcc |
| HMGB1 Box-A antagonist | HMG Biotech | HM-012 |
| LPS  Nigericin  RPMI medium | Invivogen  Cell Signaling Technology  ThermoFisher Scientific | tlrl-eklps  66419  R8758 |
| HI-FBS | Gibco | 16140-071 |
| TWEEN-20 | Sigma-Aldrich | P9416 |
| Bovine serum albumin | New England Biolabs | B9000S |
| Trypsin-EDTA (0.05%), phenol red | Gibco | 25300120 |
| penicillin-streptomycin (10,000 U/mL) | ThermoFisher Scientific | 15140122 |
| PMA | Sigma-Aldrich | P1585 |
| SmGM™- 2 Smooth Muscle Cell Growth Medium-2 SingleQuots™ supplements and growth factors | Lonza | CC-4149 |
| PMSF | Thermo Scientific | Cat#, 36978 |
| Protease inhibitor mixture | Roche | 116974980011 |
| RNeasy Micro Kit | Qiagen | 74004 |
| Cell lysis buffer 10x | Cell Signaling Technology | 9803 |
| Pierce™ co-immunoprecipitation kit | Thermo Scientific | 26149 |
| Caspase-1 Assay Kit (Fluorometric) | Abcam | ab39412 |
| Cell-proliferation ELISA, BrdU (colorimetric) | Roche | 11647229001 |
| Water, endotoxin-free | Quality Biological | 118-325-131 |
| ECL™ western blotting reagents | Sigma-Aldrich | GERPN21061 |
|  |  |  |
| **Cell lines** |  |  |
| Human THP-1 monocytes | ATCC | TIB-202 |
| Human pulmonary artery smooth muscle cells | Lonza | CC-2581 |
|  |  |  |
| **Animals** |  |  |
| C57BL/6 | Jackson Laboratory | strain #000664 |
| RELMα KO mice | Jackson Laboratory | strain #029976 |
