# Supplementary material for: Human resistin is critical to activation of the NLRP3 inflammasome in macrophages
Source: PLoS One. 2026 Apr 10;21(4):e0337682. doi: 10.1371/journal.pone.0337682 (PMC13068211; doi:10.1371/journal.pone.0337682)
Supplement: S1 Raw Images — (PDF) [file pone.0337682.s008.pdf]

Figure 2B

HMGB1

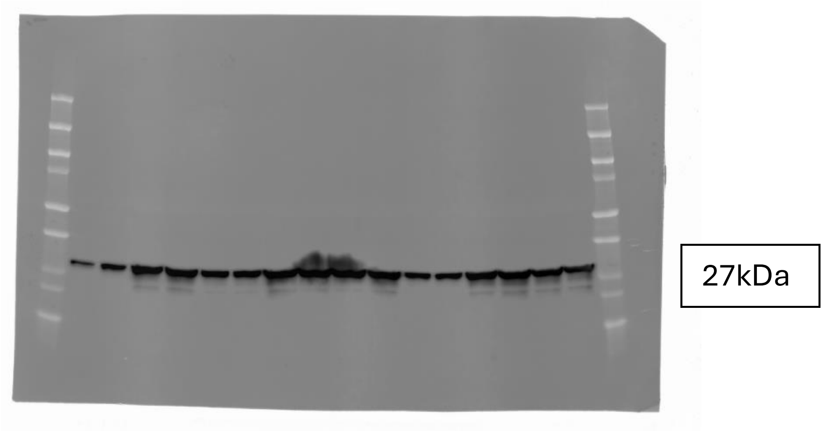

NLRP3

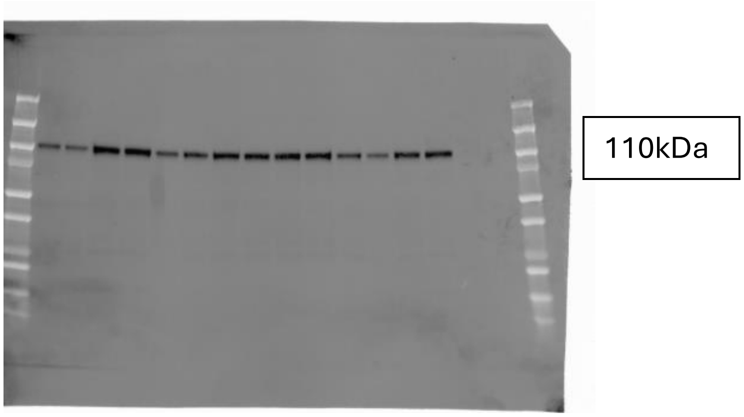

BTK

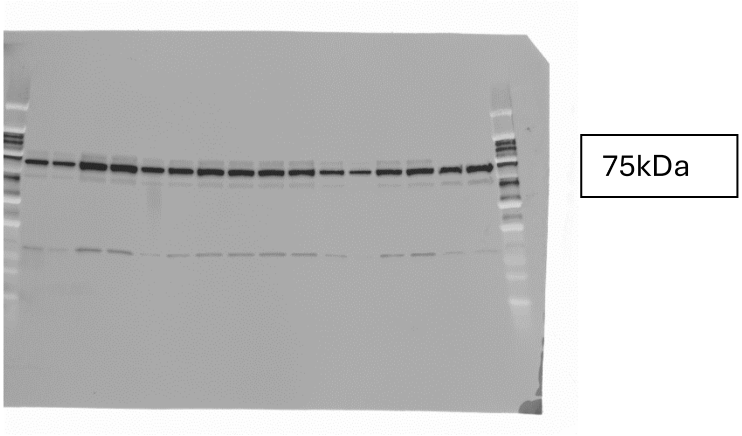

Pro-caspase-1 and Cleaved Caspase-1

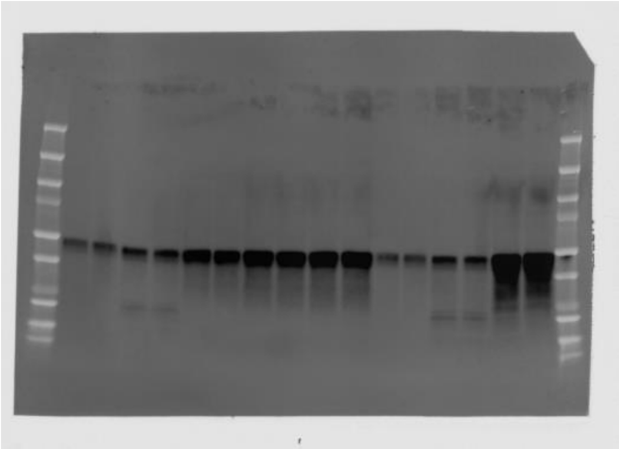

50kDa

20kDa

Pro-IL-1β and Cleaved IL-1β

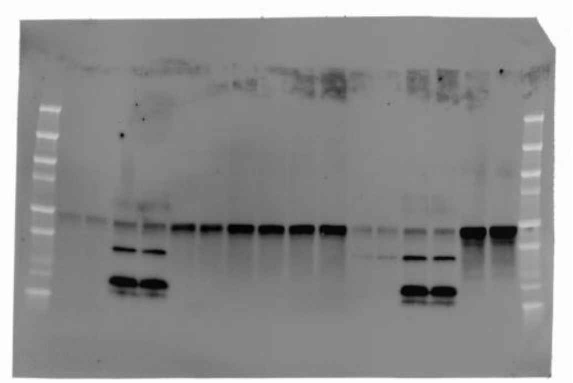

38kDa

17kDa

Pro-IL-18 and Cleaved IL-18

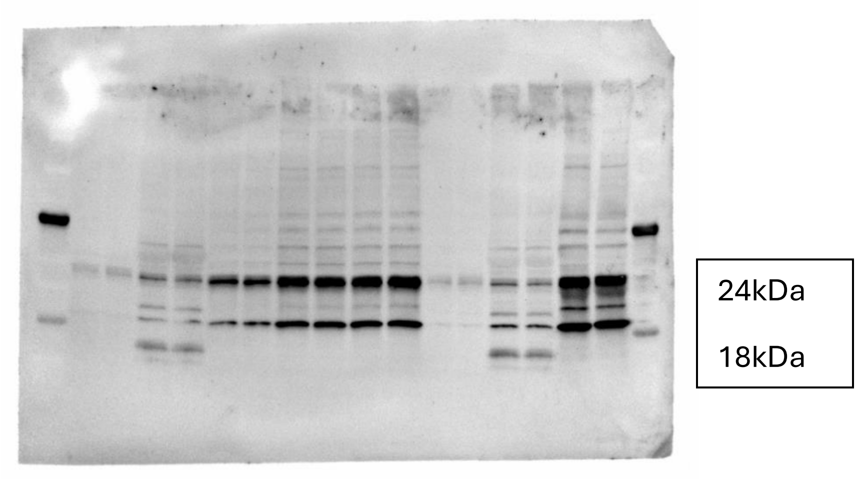

24kDa

18kDa

Beta Actin

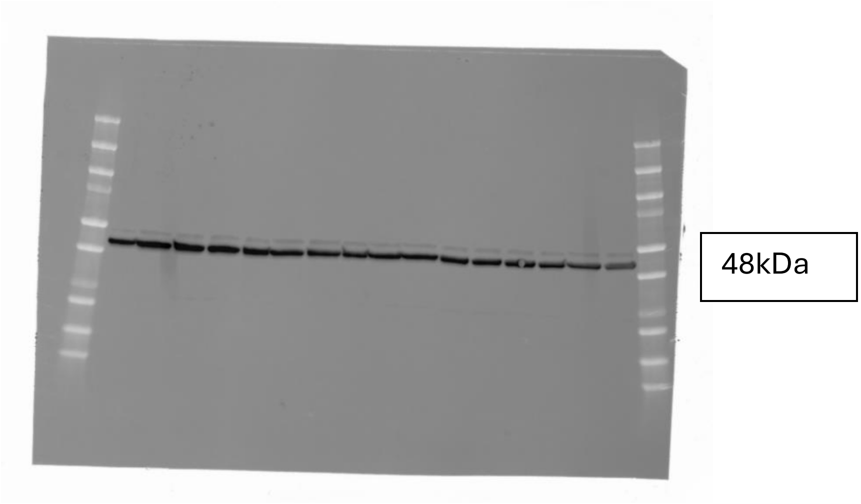

48kDa

Figure 3B

Phospho-tyrosine (P-NLRP3, P-BTK)

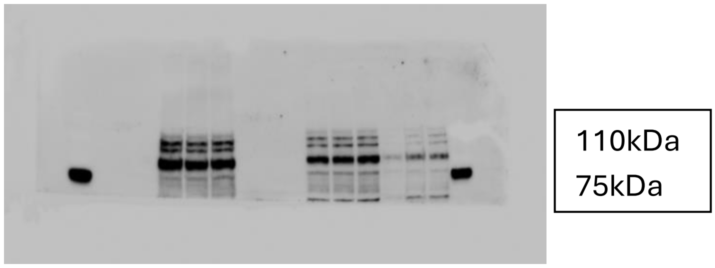

110kDa
75kDa

Total BTK

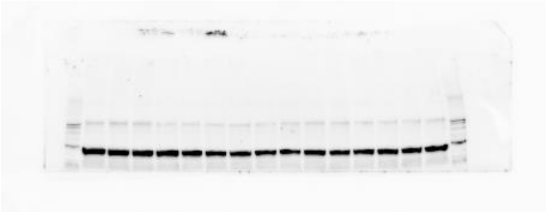

75kDa

Total NLRP3

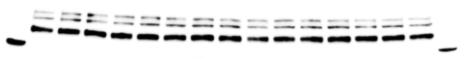

110kDa

Beta Actin

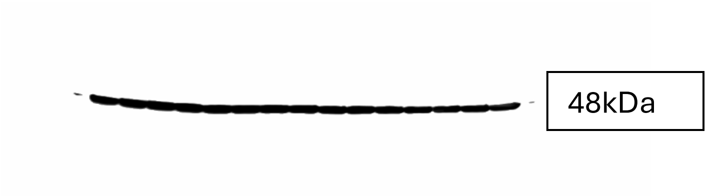

48kDa

Figure 4C

BTK

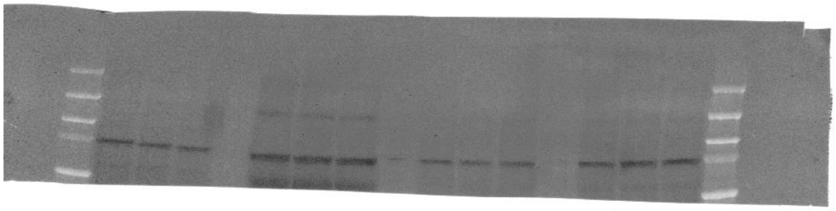

75kDa

HMGB1

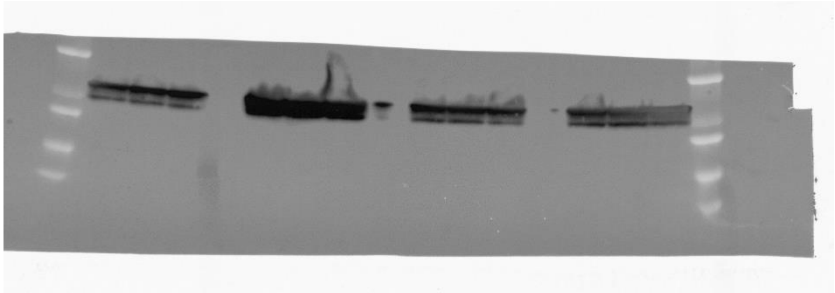

27kDa

NLRP3

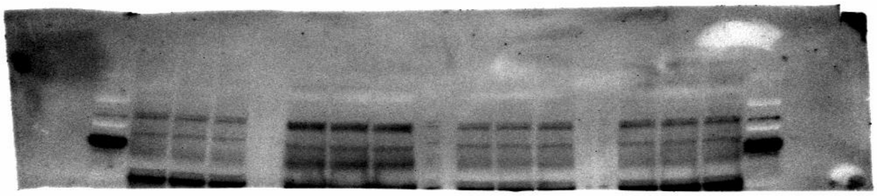

110kDa

Beta Actin

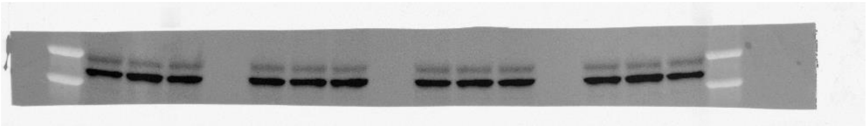

48kDa

Figure 6A

p-AKT

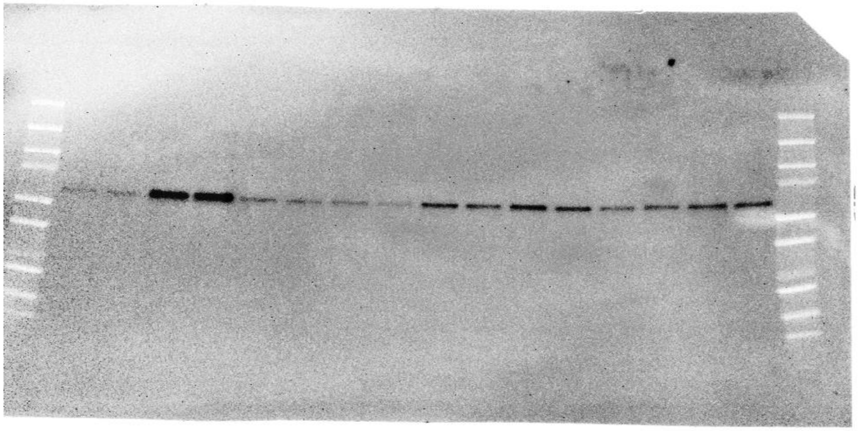

60kDa

Total AKT

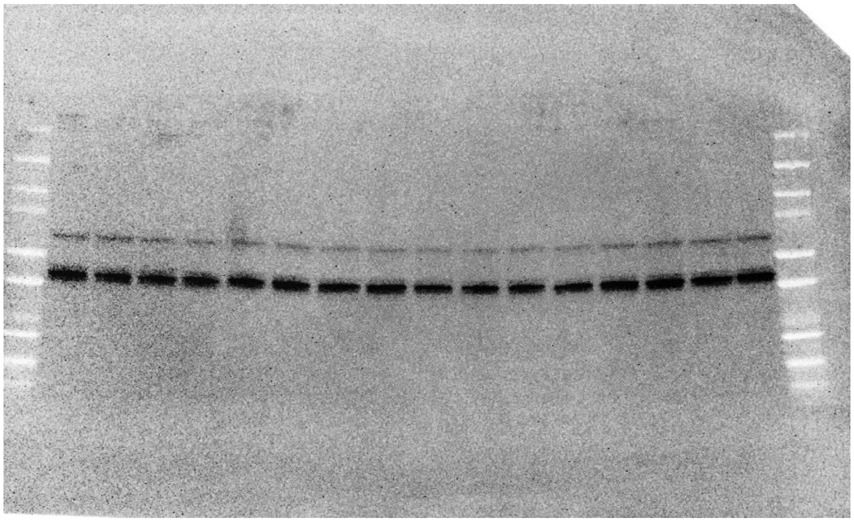

60kDa

p-ERK1/2

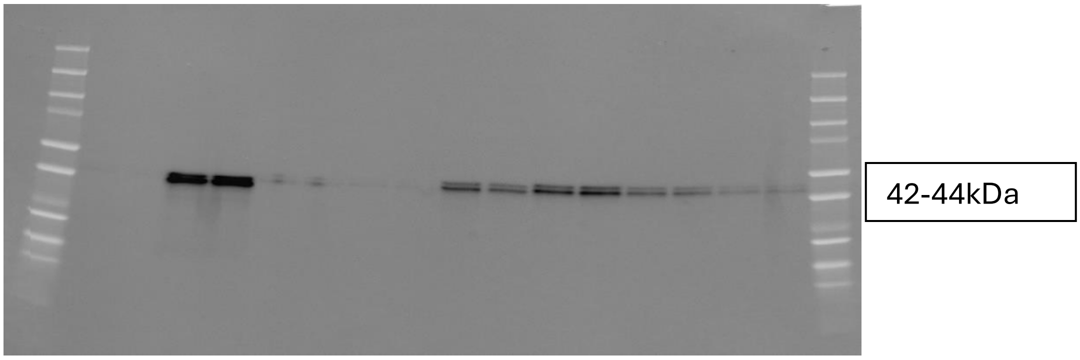

42-44kDa

Total ERK1/2

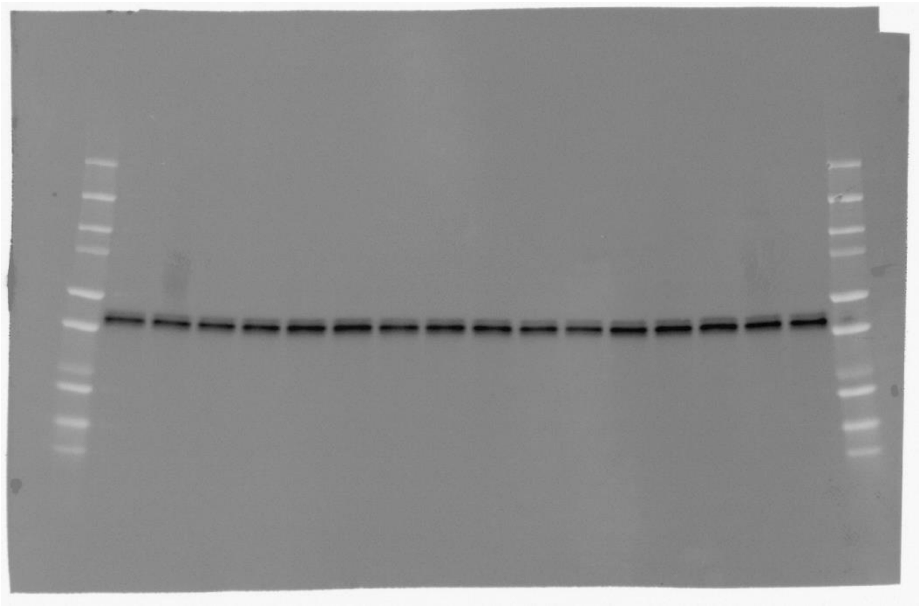

42-44kDa

Figure 6D

MMp-3

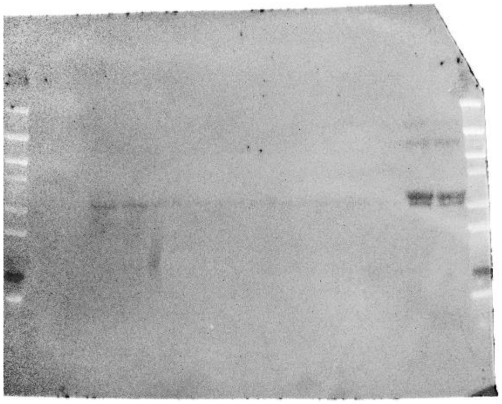

55kDa

Pre-MMp-1 and Pro-MMp-1

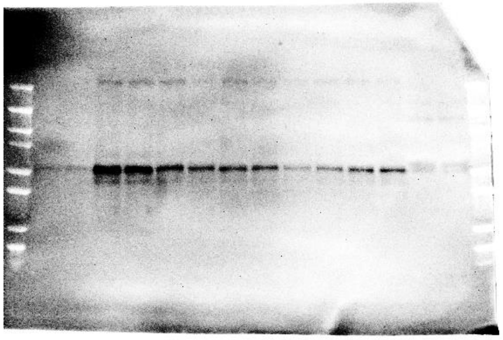

50kDa
45kDa

Beta Actin

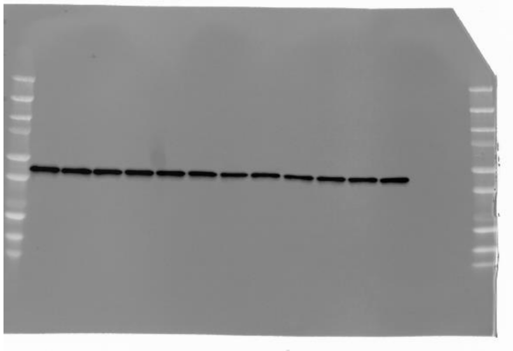

48kDa

Supplemental Figure 1A

NLRP3

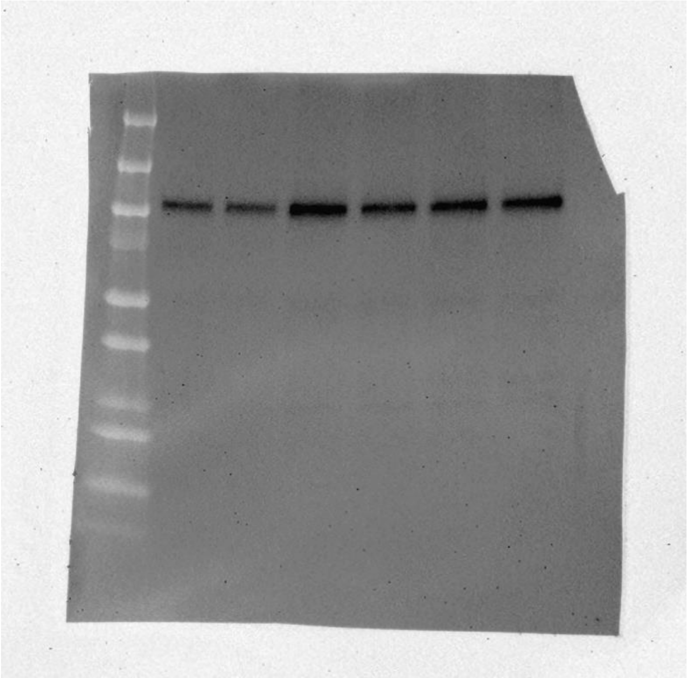

110kDa

Pro-Caspase-1

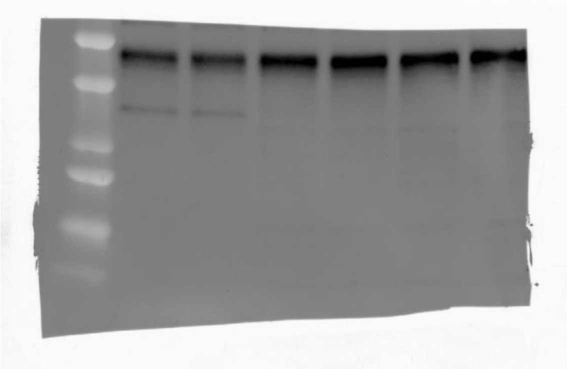

48kDa

Pro-IL-1β and Mature IL-1β

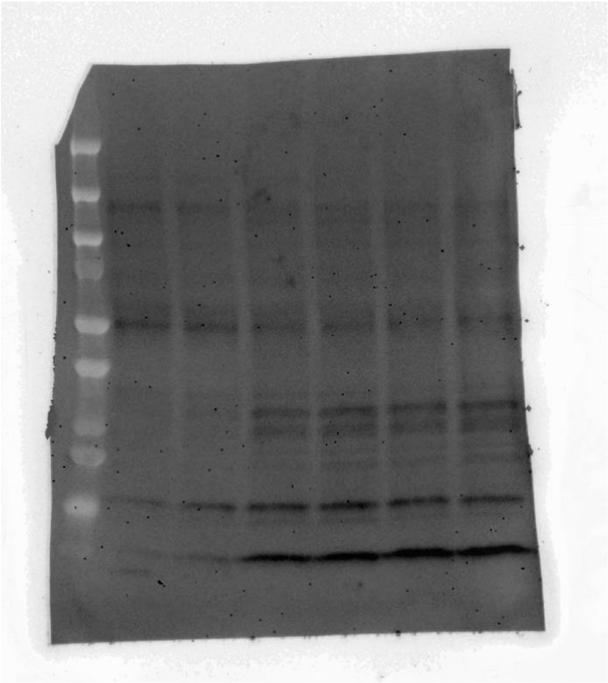

31kDa

17 kDa

Pro-IL-18 and Mature IL-18

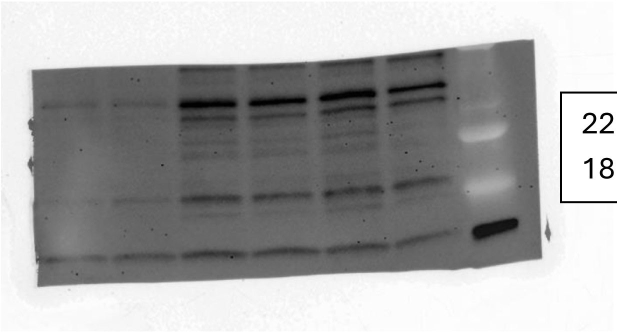

22kDa
18kDa

Beta Actin

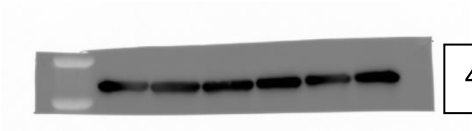

48kDa

Supplemental Figure 3B

Total BTK

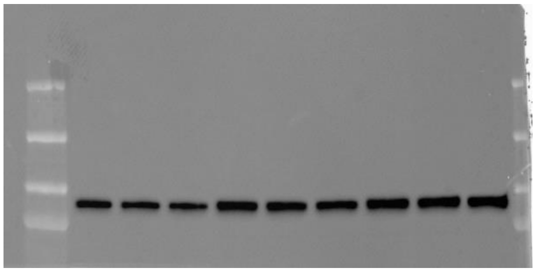

77kDa

Beta Actin

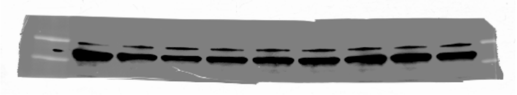

48kDa

Supplemental Figure 3D

BTK X X X X X

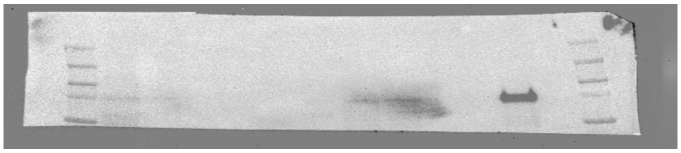

75kDa

Flag X X X X X

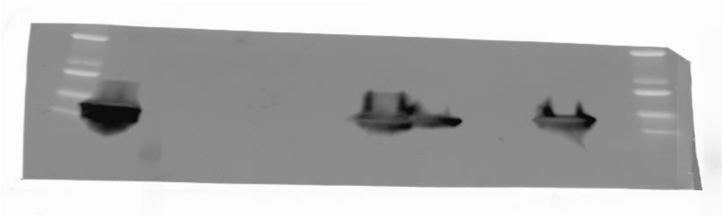

12kDa

Supplemental Figure 3E

p-BTK (Y551)

X X

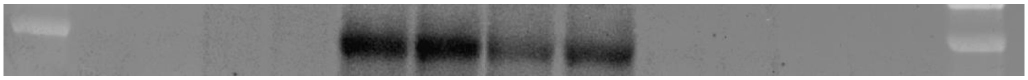

75kDa

Total BTK

X X

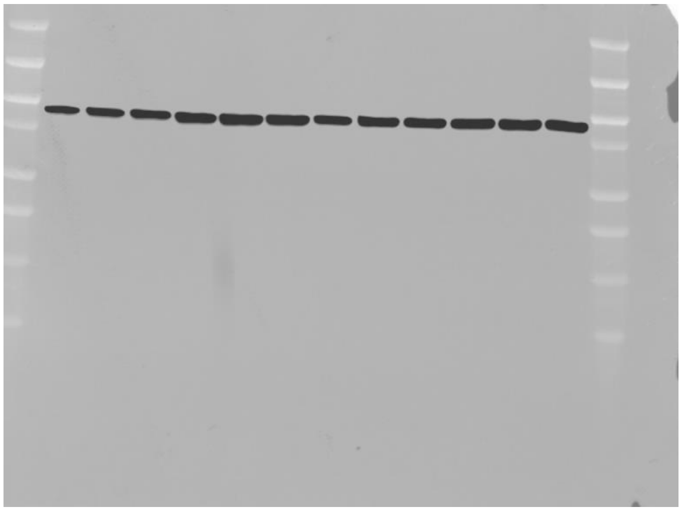

75kDa

Beta Actin

X X

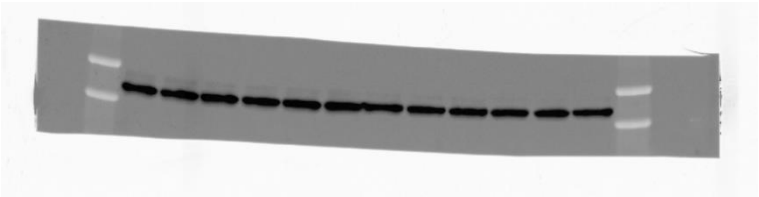

48kDa
