# Supplementary material for: Human resistin is critical to activation of the NLRP3 inflammasome in macrophages
Source: PLoS One. 2026 Apr 10;21(4):e0337682. doi: 10.1371/journal.pone.0337682 (PMC13068211; doi:10.1371/journal.pone.0337682)
Supplement: Attachment — Submitted filename: Response to Reviewers.docx [file pone.0337682.s011.docx]

**Journal Requirements:**

When submitting your revision, we need you to address these additional requirements.

1. Please ensure that your manuscript meets PLOS ONE's style requirements, including those for file naming. The PLOS ONE style templates can be found at

https://journals.plos.org/plosone/s/file?id=wjVg/PLOSOne_formatting_sample_main_body.pdf and https://journals.plos.org/plosone/s/file?id=ba62/PLOSOne_formatting_sample_title_authors_affiliations.pdf

File names have been updated to match the information in the provided links: “Response to Reviewers”, “Revised Manuscript with Track Changes”, and “Manuscript.” All section heading and font size have been ensured to match the provided templates.

Author affiliations were updated to fit *PLOS One* requirements. Figure citations were updated (e.g., from Figure 1 to Fig 1, from Supplemental Figure 1 to S1 Fig)

2. Thank you for stating in your Funding Statement:

[This work was supported by National Institutes of Health (NIH) Centers for Advanced Diagnostics and Experimental Therapeutics in Lung Diseases (CADET) I grant P50HL107182 (RAJ), NIH CADET II grant 5UH2HL123827-02 (RAJ), NIH grant R01HL138497 (RAJ), NIH grant R01HL166717 (RAJ, QL), and American Heart Association grant #938614/2022 (QL).

https://www.nhlbi.nih.gov/science/lung-diseases

https://www.nih.gov/about-nih/nih-almanac/national-heart-lung-blood-institute-nhlbi

https://www.heart.org/

The sponsors/funders did not play any role in the study design, data collection and analysis, decision to publish, or preparation of the manuscript.].

Please provide an amended statement that declares *all* the funding or sources of support (whether external or internal to your organization) received during this study, as detailed online in our guide for authors at http://journals.plos.org/plosone/s/submit-now. Please also include the statement “There was no additional external funding received for this study.” in your updated Funding Statement.

Please include your amended Funding Statement within your cover letter. We will change the online submission form on your behalf.

The following amended funding statement is in the submitted revised cover letter:

“This work was supported by the National Institutes of Health (NIH) Centers for Advanced Diagnostics and Experimental Therapeutics in Lung Diseases (CADET) I grant P50HL107182 (RAJ), NIH CADET II grant 5UH2HL123827-02 (RAJ), NIH grant R01HL138497 (RAJ), NIH grant R01HL166717 (RAJ, QL), and American Heart Association grant #938614/2022 (QL). These are all the funding and sources of support received during this study. None of the sponsors/funders played a role in the study design, data collection and analysis, decision to publish, or preparation of the manuscript. There was no additional external funding received for this study.”

3. Please include your full ethics statement in the ‘Methods’ section of your manuscript file. In your statement, please include the full name of the IRB or ethics committee who approved or waived your study, as well as whether or not you obtained informed written or verbal consent. If consent was waived for your study, please include this information in your statement as well.

The animal ethics statement was edited to “All animal experiments were used in accordance with the guidelines of the Johns Hopkins Animal Care and Use Committee.” The ethics committee is the Johns Hopkins Animal Care and Use Committee.

Lung histology (figures 3E and 5A-C) was performed on tissue slices of deceased human PH patients and controls obtained from the Johns Hopkins Pathology laboratory. We included in the Methods section under human lung autopsy “This work was approved by the Johns Hopkins Human Institutional Review Board under Exemption 4 for unidentified human tissue samples.”

4. PLOS ONE now requires that authors provide the original uncropped and unadjusted images underlying all blot or gel results reported in a submission’s figures or Supporting Information files. This policy and the journal’s other requirements for blot/gel reporting and figure preparation are described in detail at https://journals.plos.org/plosone/s/figures#loc-blot-and-gel-reporting-requirements and https://journals.plos.org/plosone/s/figures#loc-preparing-figures-from-image-files. When you submit your revised manuscript, please ensure that your figures adhere fully to these guidelines and provide the original underlying images for all blot or gel data reported in your submission. See the following link for instructions on providing the original image data: https://journals.plos.org/plosone/s/figures#loc-original-images-for-blots-and-gels.

In your cover letter, please note whether your blot/gel image data are in Supporting Information or posted at a public data repository, provide the repository URL if relevant, and provide specific details as to which raw blot/gel images, if any, are not available. Email us at plosone@plos.org if you have any questions.

We have provided the original western blot images in the file named “S1_raw_images.” We also mentioned the file name in the revised cover letter.

5. We note that Figure 5 in your submission may contain copyrighted images. All PLOS content is published under the Creative Commons Attribution License (CC BY 4.0), which means that the manuscript, images, and Supporting Information files will be freely available online, and any third party is permitted to access, download, copy, distribute, and use these materials in any way, even commercially, with proper attribution. For more information, see our copyright guidelines: http://journals.plos.org/plosone/s/licenses-and-copyright.

We require you to either (1) present written permission from the copyright holder to publish these figures specifically under the CC BY 4.0 license, or (2) remove the figures from your submission:

1. You may seek permission from the original copyright holder of Figure 5 to publish the content specifically under the CC BY 4.0 license.

We recommend that you contact the original copyright holder with the Content Permission Form (http://journals.plos.org/plosone/s/file?id=7c09/content-permission-form.pdf) and the following text:

“I request permission for the open-access journal PLOS ONE to publish XXX under the Creative Commons Attribution License (CCAL) CC BY 4.0 (http://creativecommons.org/licenses/by/4.0/). Please be aware that this license allows unrestricted use and distribution, even commercially, by third parties. Please reply and provide explicit written permission to publish XXX under a CC BY license and complete the attached form.”

Please upload the completed Content Permission Form or other proof of granted permissions as an "Other" file with your submission.

In the figure caption of the copyrighted figure, please include the following text: “Reprinted from [ref] under a CC BY license, with permission from [name of publisher], original copyright [original copyright year].”

2. If you are unable to obtain permission from the original copyright holder to publish these figures under the CC BY 4.0 license or if the copyright holder’s requirements are incompatible with the CC BY 4.0 license, please either i) remove the figure or ii) supply a replacement figure that complies with the CC BY 4.0 license. Please check copyright information on all replacement figures and update the figure caption with source information. If applicable, please specify in the figure caption text when a figure is similar but not identical to the original image and is therefore for illustrative purposes only.

Figure 5 and other graphical abstracts were made in BioRender. We have provided the publication licenses in the file named “Combined Publication Licenses.”

If the reviewer comments include a recommendation to cite specific previously published works, please review and evaluate these publications to determine whether they are relevant and should be cited. There is no requirement to cite these works unless the editor has indicated otherwise.

**Review Comments to the Author**

Please use the space provided to explain your answers to the questions above. You may also include additional comments for the author, including concerns about dual publication, research ethics, or publication ethics. (Please upload your review as an attachment if it exceeds 20,000 characters)

**Reviewer #1**: Dear Dr. Roger A Johns.
I would like to thank you for the opportunity to review this excellent, meticulous, and well-planned work. The results are described meticulously and elegantly.
My only concern is regarding the manipulation of the western blot images. Unfortunately, the resolution of images 2, 3, 4, and 6 in the manuscript is not detailed enough to allow for a decent review.

Thank you for your review and pointing out these issues. We too had issues with the resolution in the pdf version of the manuscript. The TIF file when downloaded should provide a clearer resolution. Additionally, we have provided our original western blot images in supplemental file 1 (S1_raw_images). Most of them consist of uncut membranes. Membranes that were cut prior to imaging were done so to prevent previously imaged proteins from the same membrane from masking the signals of the new protein.

However, in image S2, panels B, D, and E, which show western blot images, there are some concerning features, such as anomalous uniformity in backgrounds and load controls, as well as possible excessive background correction and assembly of independent gels.
Is it possible to obtain the original unedited files (raw tiff files for each membrane), full exposure (“full uncut blots”), and acquisition metadata from you?
This would give me peace of mind regarding such beautiful research work.

Thank you for pointing out these issues in S2 Fig (now S3 Fig). For panel B, the extra bands are from the heavy chains from our secondary antibody. We had used a mouse host for our primary antibody, so the band above beta actin (~50kDA) was the heavy chain. For panel D, there were oversaturation present when imaging, resulting in the anomalous uniformity. Unfortunately, this was a common occurrence when conducting Co-IP experiments. We provided the uncropped membrane images in supplemental file 1 (S1_raw_images) for the full membrane. Of note, we cropped this image for panel D due to the large amount of unused membrane between the input and elution lanes. For panel E, we’ve edited our assembly to provide a more uniform image of P-BTK (Y551). Additionally, we have added the uncropped image in supplemental file 1. Due to previous protein signals masking the phosphorylated BTK (Y551), we had to cut the membrane prior to imaging

**Reviewer 2**

The manuscript by Kariyawasam et al. investigates human resistin (hResistin) and its rodent homolog, resistin-like molecule alpha (RELMα), as upstream regulators of NLRP3 inflammasome priming and activation in macrophages. They act via HMGB1–NF-κB–dependent transcriptional priming and BTK-mediated phosphorylation pathways, respectively. The study demonstrates that hResistin induces HMGB1 release to prime NLRP3 and its downstream pro-cytokines, engages BTK to promote NLRP3 tyrosine phosphorylation, caspase‑1 activation, and maturation of IL‑1β and IL‑18, and drives PVSMC proliferation through these cytokines, with upregulation of this axis confirmed in hypoxic mouse lungs and pulmonary hypertension patient tissues, and loss of hypoxia-induced signalling in RELM-deficient mice. The following major and minor issues should be resolved before the manuscript is suitable for publication.

**Major comments**

1. qRT‑PCR data are presented at 24 h and described as assessing pro‑IL‑1β, pro‑caspase‑1, and pro‑IL‑18 “forms,” but mRNA measurements reflect gene expression and cannot distinguish zymogen versus cleaved protein species; only immunoblotting and ELISA can discriminate inactive precursors from active cytokines, and gene expression kinetics should be evaluated at earlier time points distinct from protein analyses to avoid misinterpretation and overstatement throughout the text.

Thank you for your review and pointing out these issues. We agree that the mRNA measurements cannot distinguish between zymogens and cleaved proteins. We removed ‘pro-’ from the associated figures to reflect this. We also conducted time course qPCR experiments with the same groups at 6, 12, and 18 hours to strengthen our data, with the results following a similar trend to the 24 hour treated groups. Additionally, we conducted ELISAs with the same groups, including a positive control of LPS plus nigericin to quantify the cleaved IL-1β and IL-18 (supplemental figure 4).

2. The study does not include a canonical positive control for NLRP3 inflammasome activation, such as LPS priming followed by ATP or nigericin stimulation, which is necessary to benchmark hResistin/RELMα-induced responses against established NLRP3 activation paradigms.

Thank you for pointing out these issues. We agree that having a canonical positive control for NLRP3 inflammasome is critical to benchmark hResistin induced responses. To improve our data. We added in supplemental figures 1A and B to show western blot images and comparisons between the control, hResistin-treated, and LPS and nigericin treated THP-1 derived macrophages. Using LPS and nigericin at concentrations of 100ng/mL and 10µM, respectively (similar to reference 5; PMID:34554188), we found comparable effects in protein expression for NLRP3, pro-caspase-1, total IL-1β, and total IL-18 (supplemental figure 1A and B).

3. The authors state that both canonical and non-canonical inflammasome pathways and their downstream signalling are examined, but experimental evidence distinguishing these pathways is not clearly defined or systematically presented.

Thank you for pointing out this issue. Since our experimental evidence surrounds the activation of caspase-1 via NLRP3, the pathway we are exploring is the canonical pathway. To alleviate confusion, we added in the introduction that we were exploring the canonical pathway.

4. The graphical abstract depicts multiple cytokines, yet the manuscript does not comprehensively measure or report several of these mediators, rendering the schematic potentially misleading and requiring alignment of depicted factors with experimentally quantified analytes.

Thank you for pointing out this issue. The main cytokines in the graphical abstracts were hResistin/RELMα, HMGB1, IL-1β, and IL-18. One major interest in this manuscript was to ascertain the role of hResistin in priming the NLRP3 inflammasome through HMGB1. Since we were directly stimulating the cells with hResistin, we didn’t measure its levels in the supernatant. We have previously published work that goes more in depth regarding hResistin and HMGB1’s relationship (reference 10, PMID: 31597444 and reference 11, PMID: 31611261). To strengthen our data surrounding the latter two cytokines, we measured their levels in the supernatant of THP-1 differentiated macrophages (supplemental figure 4). Although we didn’t directly measure the expression of IL-1β’s receptor (IL-1R), previously published works from others have identified the receptor and its agonist as potential primers of the NLRP3 inflammasome (reference 3, PMID: 31036962; reference 37, PMID: 31284572). To alleviate confusion, we removed IL-1R from the graphical abstracts and added in the ‘Discussion’ section the potential role of IL-1β acting as an endogenous cytokine that can prime the inflammasome.

5. The overall design links in vitro macrophage signalling to in vivo pulmonary hypertension models; however, the 4-day hypoxia protocol in mice likely captures early inflammatory and remodelling events rather than fully established chronic pulmonary hypertension, which limits direct translational extrapolation to long-standing human disease.

Thank you for pointing out these issues. We agree that the 4-day hypoxia model captures early inflammatory and remodelling events. We and others doing resistin research believe that the role of resistin is in early inflammatory responses rather than in the late stages. The main purpose of this study was to understand the role of resistin in early immune responses that causes and develops chronic hypoxia at late stages. Indeed, we find that in more extended rodent models of PH (2, 3, or 6 weeks), RELMα expression is significantly decreased; however, human expressions of hResistin appears differently than rodent models in the long term. In our human PAH biomarker study, we found hResistin to be elevated throughout the course of the disease and correlated with more rapid progression to severe disease and death (reference 45, PMID: 38844967).

6. Phospho‑tyrosine immunoblotting is interpreted as specific evidence for BTK- and NLRP3-directed phosphorylation but lacks site specificity; BrdU incorporation assays quantify PVSMC proliferation but do not assess migration despite claims involving MMP-1-driven motility; and no cytokine ELISA data are provided to quantify IL-1β and IL-18 secretion into supernatants.

Thank you for pointing out these issues. We agree that the use of a phospho-tyrosine antibody lacks site specificity. Using the same groups as figure 2B, we showed specific phosphorylation of BTK at site tyrosine 551 in supplemental figure S3E and F. Unfortunately, there are no commercially available site specific NLRP3 phosphorylation antibodies. To overcome this, we blotted total NLRP3 on the same membrane after the phospho-tyrosine antibody was used, which resulted in matching molecular weights. This experimental method is similar to that of Bittner *et al*’s paper (PMID: 34554199), where they have shown NLRP3 activation by BTK using co-IP and immunoblotting with phosphotyrosine and NLRP3 antibodies. We didn’t conduct site-specific loss of function experiments as they were out of the scope of our work, and we were interested in the upstream regulation of BTK by hResistin. As for the migration assays pertaining to MMP-1 driven motility, we removed ‘migration’ from the figure captions to fit the data shown. Additionally, we are currently planning to do further investigations to understand the detailed mechanism and supplement our past publications pertaining SMC migration (reference 8, PMID: 21139050)

7. The experimental framework appropriately incorporates pharmacological (ibrutinib, MCC950) and genetic (NLRP3 knockout, RELM knockout) controls, and the use of PMA‑differentiated, rested THP‑1 macrophages reduces PMA‑related bias; nonetheless, the co‑immunoprecipitation strategy relies on FLAG‑tagged recombinant hResistin incubated with lysates, which may generate non‑physiological interactions and should be complemented by approaches that detect endogenous complexes in situ (e.g., proximity ligation assays or native co‑IP).

Thank you for pointing out these issues. In figure 3E, we saw colocalization of BTK with hResistin in patients with PH. To supplement this, we conducted Co-IP experiments at 4°C, which according to manufacturer instructions, allows the protein complexes to remain in its native state. Additionally, since we wanted to show that hResistin binds to BTK, we didn’t want to use anti-BTK or anti-hResistin antibodies with the coupling resins as it’s unclear whether the antibodies would inhibit the binding between the two proteins. To overcome this, we used a FLAG-tagged recombinant hResistin and used an anti-FLAG antibody with the coupling resin to bind to the hResistin.

8. The 4-day hypoxia model robustly induces inflammatory signalling but does not establish full pulmonary hypertension physiology, as right ventricular systolic pressure, right ventricular hypertrophy, and fibrosis endpoints are not reported, while autopsy lung samples from PH patients represent heterogeneous, end-stage disease with non‑standardized treatment and comorbidity histories.

Thank you for pointing out these issues. We agree that the 4-day hypoxia model captures the early inflammatory and remodelling events while being too early to establish full PH physiological markers. The main purpose of this study was to understand the role of hResistin in early immune responses that causes and develops chronic hypoxia at late stages. Previous explorations found a strong correlation between hResistin serum levels and pulmonary arterial hypertension severity (reference 45, PMID: 38844967), suggesting hResistin may be a marker to predict disease progression. To supplement this finding, we wanted to understand the time point where we should treat and block resistin to have better physiological meaning, which is why we chose the 4-day hypoxia model (to capture early events of hypoxia-induced PH).

9. The data are used to argue that hResistin is an upstream therapeutic target for NLRP3 inhibition via monoclonal antibody blockade, but phospho‑tyrosine blots are overinterpreted as mapping to specific BTK and NLRP3 residues without corroborating evidence from site-directed mutagenesis or mutational loss-of-function studies.

Thank you for pointing out these issues. We agree that the phospho-tyrosine antibody lacks site specificity when examining hResistin as an upstream target of NLRP3. To add specificity using the same groups, supplemental figure 3E and F show BTK phosphorylation at tyrosine site 551. Unfortunately, there are no commercially available site specific phosphorylated NLRP3 antibodies. To overcome this, we blotted total NLRP3 on the same membrane that was used for the phospho-tyrosine image and the molecular weights lined up. We didn’t pursue site-directed mutagenesis or mutational loss-of-function as Bittner *et al.* (reference 5, PMID: 34554188) have examined BTK’s role in phosphorylating NLRP3. Our work here pertains more to the upstream regulation of BTK by hResistin.

10. The main conceptual innovation is the integration of hResistin/RELMα into both the priming and activation phases of NLRP3 signaling, extending prior work on BTK–NLRP3 regulation; however, mechanistic depth is limited regarding HMGB1–NF‑κB–RAGE/TLR4 circuitry, ASC speck formation, and upstream NF‑κB activation dynamics in this context.

Thank you for pointing out these issues. We agree that a detailed mechanism needs to be further investigated and are planning experiments for a future publication. We have previously published the role of hResistin and how it affects the HMGB1/RAGE pathway in pulmonary hypertension (reference 10, PMID: 31597444 and reference 11, PMID: 31611261).

11. Important gaps remain, including the absence of direct assays of NLRP3 oligomerisation and ASC polymerisation (e.g., ASC cross-linking or speck imaging) and the lack of controls for alternative BTK activators (such as TLR ligands), which may confound attribution of BTK activation solely to hResistin/RELMα.

Thank you for pointing out these issues. We agree that direct assays of NLRP3 oligomerization and ASC polymerization can be used to show activation of the NLRP3 inflammasome complex, and we are planning to conduct these experiments for another publication in the future. To circumvent this, we used an assay that can directly measure caspase-1 activation (figure 3C). Together, with figure 2B showing that MCC950 inhibited the cleavage of pro-caspase-1, the NLRP3 inflammasome (which includes ASC) plays a role in the activation of caspase-1.

12. Although RELM knockout abolishes hypoxia-induced HMGB1, BTK, and NLRP3 upregulation in mouse lungs, alternative explanations persist because HMGB1 can signal through multiple receptors, including TLR4 and RAGE, which are not dissected experimentally in this study.

Thank you for pointing out these issues. We agree that a detailed mechanism of HMGB1 with its multiple receptors and downstream pathways needs to be further investigated. The novelty of this paper surrounds hResistin’s ability to induce HMGB1 secretion, which primes the NLRP3 inflammasome formation. We have previously studied the role of hResistin in the HMGB1/RAGE pathway in pulmonary hypertension (reference 10, PMID: 31597444 and reference 11, PMID: 31611261).

13. The proposed hResistin–BTK–NLRP3 axis would be substantially strengthened by validation in primary human macrophages, by genetic silencing (siRNA/shRNA) of hResistin and BTK, and by visualisation of NLRP3–ASC specks to demonstrate bona fide inflammasome assembly.

Thank you for pointing out these issues. We agree that the axis would be strengthened using primary human macrophages. We are currently performing experiments using primary macrophages isolated from PBMCs and using siRNA to examine the axis at a deeper level for a future publication.

**Minor comments**

1. Nomenclature and labelling should be standardised: for example, “hResistin” is used in the text, whereas figure panels and the graphical abstract sometimes show “Hresistin”; a single, consistent designation should be applied throughout.

Thank you for pointing out these inconsistencies. We have gone through the figures and revised them to be hResistin when applicable (Figure 1, 2A, 3E, 4A, 5B, 6F, S3A, and S3E).

2. Several textual elements require correction and harmonisation, including “pro‑caspase‑1” to “pro‑caspase‑1” in the Abstract and consistent ibrutinib dosing units in the Methods section (currently reported as 100 ng/mL and 60 μM).

Thank you pointing out these issues. In the methods, we made sure to change our dosing units of ibrutinib to be 100ng/mL; however, we’re unsure what was meant by the change from “pro-caspase-1” to “pro-caspase-1” as the text on our end appears the same. We did review the manuscript to ensure we specify that the pro form of caspase-1 is the one being cleaved.

3. Key references need to be specified: the BTK–NLRP3 tyrosine phosphorylation literature (currently cited broadly as references 5–7) should clearly identify the modified residues, and primers listed in Supplementary Table 1 should be quantitatively integrated into the main Results (e.g., fold‑change ranges and statistics).

Thank you for pointing out these issues. In the introduction we separated reference 5 from 6 and 7 as reference 5 refers more to the specific NLRP3 phosphorylation sites while 6 and 7 focus more on BTK and its tyrosines. We didn’t want to identify the exact modified residues on NLRP3 as it was beyond our scope of work. Additionally, we went back into the main results to quantitatively discuss specific fold change increases or fold change ranges, depending on the context.

4. Several figure-related issues require clarification: Fig. 2C is annotated as n=9, but bar appearance suggests fewer replicates; Fig. 3B phospho‑tyrosine bands are not annotated with molecular weights; and Fig. 6D refers to “MMP-driven migration,” although only MMP‑1 protein levels are shown without accompanying migration assays.

Thank you for pointing out these issues. We went back through our figure captions to correct these errors. Figure 2B now shows n=10 for the western blot analysis, with n= 6 for the NLRP3 KO cells, to reflect the bar appearance. Additionally, we added in the appropriate molecular weights to figure 3B, 6A, S3A, and S3D. We agree that MMP-driven migration needs to be further investigated. To alleviate confusion, we removed migration from the figure caption to better match the data shown. Additionally, we are currently planning to do further investigations to understand the detailed mechanism and supplement our past publications pertaining SMC migration (reference 8, PMID: 21139050)

5. Abbreviations and numerical entries are sometimes inconsistent or contain typographical errors, including variable use of “pro‑IL‑1” versus “pro‑interleukin‑1” and apparent formatting issues in Table 1 hemodynamic data (e.g., “31.51” for cardiac output/cardiac index).

Thank you for pointing out these issues. After examining the manuscript, we made sure we only used ‘interleukin’ when mentioning it for the first time in the abstract and introduction. After words, we stuck with using ‘IL’ as the abbreviations. The only other use of ‘interleukin’ outside of the abstract and introduction that we could find were in figure captions, where we used them in a list of abbreviation explanations. Re-examining table 1 hemodynamic data, we made sure the information is listed as 3/1.51 and not 31.51.

6. Supplemental Figures 1–3 are cited but not fully described in the main text, and some immunohistochemistry panels lack explicit scale bars, particularly in Figure 5, which should be corrected for interpretability.

Thank you for pointing out these issues. We went back into our main results to describe these supplemental figures in more detail. We also updated our immunohistochemistry panels to ensure explicit scale bars were present for those surrounded by different magnifications (e.g., figure 5B 200x). We made sure to place these scale bars in a way to not obscure too much of the images.

7. Specific English and grammar revisions are recommended, for example:
   - “hResistins many inflammatory effects contribute to the progression of these diseases remain poorly understood” should be revised for clarity (e.g., insertion of appropriate punctuation and verb agreement).

Thank you for pointing out these issues. We went back through the manuscript to ensure the writing is clear. The example pointed out here was missing an apostrophe between ‘hResistin’ and ‘s’. With it, the verb agreement should be correct with the plural inflammatory effects agreeing with the verb form ‘contribute.’

- “pro‑caspase‑1, pro‑interleukin‑1, and pro‑IL‑18” should be presented consistently with standard cytokine nomenclature.

Thank you for pointing out these issues. As mentioned in minor comment 5, we made sure our use of ‘interleukin’ remained only in the abstract, introduction, and figure captions. Otherwise, we used the abbreviation ‘IL.’

- “causes the kinase to autophosphorylate, allowing BTK to phosphorylate NLRP3, leading to its assembly...” could be streamlined to avoid repetition and clarify sequence.

Thank you for pointing out these issues. To clarify the sequence, we opted to split this sentence in the abstract in two, separating BTK’s autophosphorylation from BTK’s phosphorylation of NLRP3, which results in its assembly.

- “was linked to vascular remodelling pathways” should be rewritten to specify the type of linkage and supporting data.

Thank you for pointing out these issues. We reworded this sentence in the abstract to specify RELMα as being linked to vascular remodelling pathways.

8. Some supplemental figures lack error bars, and there are discrepancies between n values stated in the main figures (e.g., Fig. 2C, n=9) and those in corresponding supplemental data (e.g., Supplementary Fig. 1, n=5), which should be reconciled.

Thank you for pointing out these issues. We resolved the inconsistencies in sample size in the supplemental figures’ captions. In terms of error bars, the ones that appear missing (particularly the control groups) are due to the individual data points obscuring them rather than being absent.

9. Figure formatting should be globally harmonised, including consistent representation of fold change (use either “FC” or “Fc” uniformly), ordering and labelling of panels, and alignment of panel titles and legends to reduce confusion for readers.

Thank you for pointing out this issue. We changed supplemental figure S3A’s y-axis to ensure consistency of ‘FC.’
